# Soft-MS and Computational Mapping of Oleuropein

**DOI:** 10.3390/ijms18050992

**Published:** 2017-05-06

**Authors:** Luigi Gentile, Nicola A. Uccella, Ganapathy Sivakumar

**Affiliations:** 1 Chemistry and Chemical Technology Department, University of Calabria, P. Bucci 12C, 87036 Rende, Italy; luigi.gentile@biol.lu.se; 2 MEMEG, Department of Biology, Lund University, 223 62 Lund, Sweden; 3 DIMEG Department, University of Calabria, P. Bucci 42C, 87036 Rende, Italy; nicola.uccella@unical.it; 4 IRESMO Foundation Group, via Petrozza 16A, 87040 Montalto Uffugo, Italy; 5 Department of Engineering Technology, College of Technology, University of Houston, Houston, TX 77204, USA

**Keywords:** biophenols, β-glucosidase, hydroxytyrosol, olive oil, secoiridoid, table olives, tyrosol

## Abstract

Olive oil and table olives are rich sources of biophenols, which provides a unique taste, aroma and potential health benefits. Specifically, green olive drupes are enriched with oleuropein, a bioactive biophenol secoiridoid. Olive oil contains hydrolytic derivatives such as hydroxytyrosol, oleacein and elenolate from oleuropein as well as tyrosol and oleocanthal from ligstroside. Biophenol secoiridoids are categorized by the presence of elenoic acid or its derivatives in their molecular structure. Medical studies suggest that olive biophenol secoiridoids could prevent cancer, obesity, osteoporosis, and neurodegeneration. Therefore, understanding the biomolecular dynamics of oleuropein can potentially improve olive-based functional foods and nutraceuticals. This review provides a critical assessment of oleuropein biomolecular mechanism and computational mapping that could contribute to nutrigenomics.

## 1. Introduction

**Figure 1:**
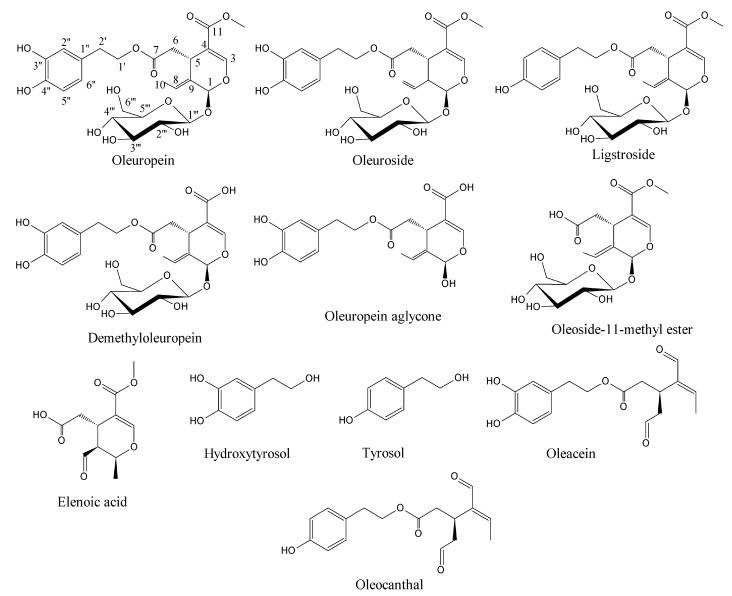
Molecular structures of olive biophenol secoiridoids.

Olive oil is extracted from olive drupes which have several health benefits and is one of the stable bioactives in the Mediterranean diet [1]. The green olive drupes are rich in biophenol secoiridoids such as oleuropein, demethyloleuropein, ligstroside, and their hydrolytic derivatives such as oleuropein aglycone, elenolate, oleoside-11-methyl ester, elenoic acid, hydroxytyrosol, and tyrosol (Figure 1) [2]. Oleocanthal and oleacein present in virgin olive oil that are dialdehydic isomeric forms of ligstroside and oleuropein aglycones, respectively, which have anticancer properties [3,4]. These are biosynthesized as combomolecules between the functional groups of biophenol secoiridoids (BPsecos). They are characterized by a chimeric structure with two chiral centers, C1 and C5 in the secoiridoid moiety. Their glucoside functionality is released via specific enzyme action by native β-glucosidase but is resistant to acidic environment [5,6]. This could result from the extended delocalization of molecular orbitals, which decreases the basicity of the glucosidic oxygen against its protonation and the facile aglycone release. The first concerted step of hydrolysis is by the native β-glucosidase. This is assisted by the enzymatic catalysis exerted onto the glucosidic oxygen. Moreover, these BPsecos are more effective than either of the separate moieties, each characterized by radical reactivity from hydroxyaromatic groups and by competing polar reactions from the secoiridoid moiety after aglycone release. Oleuropein and its isomeric oleuroside are biosynthesized via loganin route linked to the hydroxytyrosol, while in ligstroside analogue, the BP moiety is tyrosol. Hydroxytyrosol and tyrosol are also bioactives of extra virgin olive oil and table olives, which could prevent metabolic syndrome and inhibit the paracrine regulation of tumor necrosis factor-α-induced endothelial cell migration via reduced glioblastoma cell cyclooxygenase-2 expression [7,8].

Thus, significant efforts have been devoted to the investigation of these olive chimeric bioactives, which incorporate the key features of free radical quencher in the catechol moiety of the hyroxytyrosol esterified to the oleoside-11-methyl ester and are active for the polar biomolecular dynamics. This is the seco-precursor of oleacein and oleocanthal. Their dialdehydic carbonyl groups along with the *o*-quinone after the free radical reaction of hydroxytyrosol, are effective sites for DNA alkylation, protein arylation and denaturation, induced by catecholase and β-glucosidase reactions [9,10,11]. However, hydroxytyrosol and oleoside-11-methyl ester are stored in the phytoanticipin, the inactive and safe form of BPseco, stabilized as glucoside to increase solubility. BPsecos revealed stable to acid because of their chimeric structure, but less to base due to catechol and phenol units. These are compartmentalized in the vacuoles of olive mesocarp cells, while the β-glucosidase is kept in chloroplasts [12,13]. Analytical methodologies have provided information into the molecular reactivity of oleuropein [14,15,16,17,18]. Recent transcriptomic, stem cells, and clinical data suggests that oleuropein could prevent obesity, osteoporosis, and neurodegeneration [19,20,21]. It can be potentially standardized in Mediterranean functional foods and used in nutraceuticals [22]. Enhancement of bioactives in olive oil and table olives is a growing area of interest in nutrigenomics with requiring doses of antioxidants like hydroxytyrosol and tyrosol [23,24,25]. Therefore, oleuropein has been thoroughly evaluated and quantified as precursor of its hydrolytic bioactives [26,27,28]. Biomolecular approaches exploiting complex hyphenated high performance liquid chromatography electron spray ionization mass spectrometry (HPLC-ESI-MS) were used for these natural bioactive assessments [29]. The goal of this review is to highlight biomolecular dynamics and computational mapping of oleuropein.

## 2. Molecular Dynamics of Oleuropein

**Figure 2:**
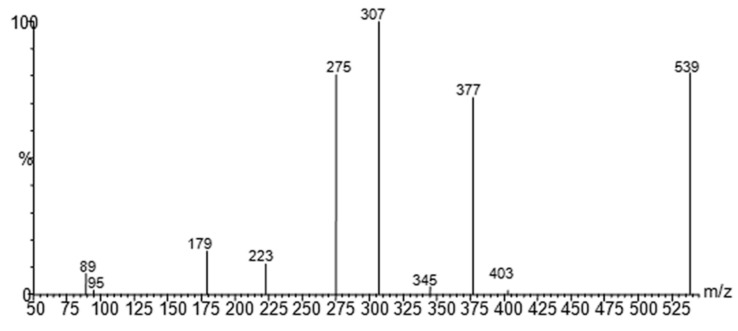
Negative ion ESI-MS/MS spectra of oleuropein [17].

**Figure 3:**
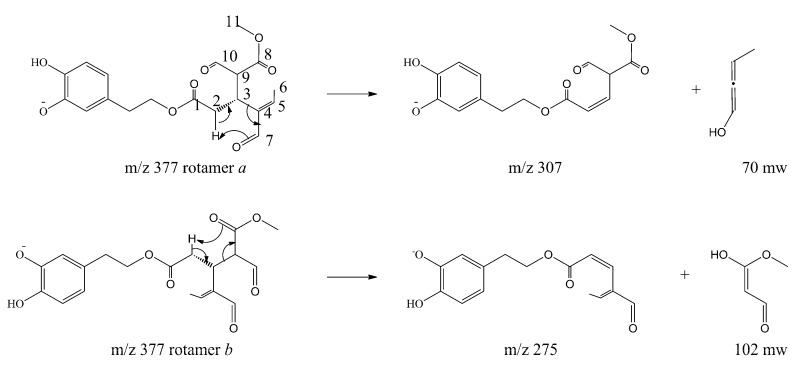
Collision activated dissociations (CAD) dissociations of oleuropein pseudomolecular anion, *m*/*z* 539.

The molecular mechanism of the olive BPseco was examined under ESI-MS and the MS/MS analysis by collision activated dissociations (CAD) [17]. The p*K*_1a_ 9.25 and p*K*_2a_ 13.00 of catechol moiety, p*K*_1a_ = 9.10 and p*K*_2a_ = 9.92 measured values, deserved the negative mode under soft ionization conditions at −28 V [30,31]. These are more sensitive than positive ionization by 20–50 fold, even if sometimes used for quantitation [32,33,34,35]. Under soft ionization experiments, oleuropein resulted in the formation of *m*/*z* 539, a pseudomolecular ion, as the lonely base peak of the ESI-MS spectra, while the CAD product ions were abundant (Figure 2). Under CAD experiments, oleuropein-H^−^, *m*/*z* 539, base peak being *m*/*z* 307, 79%, dissociated to *m*/*z* 377, oleuropeinenal-(*E*)-enol-H^−^, and the neutral glucose-H_2_O, mw = 162. A 1,5-H shift at a six member transition state is favorable for the quasi-aromatic array of three electron pair synchronous reorganization (Figure 3). Instead, BPseco + H^+^, *m*/*z* 541, revealed small pseudomolecular-ions, competitively fragmenting to analog *m*/*z* 379 and to the base peak *m*/*z* 361 in positive mode and fast atom bombardment (FAB) spectra [32,34]. Since pseudomolecular ions populated highly energized levels, protonation on different basic sites controlled the overall reactivity gas phase oleuropein+H^+^. In fact, glucose was eliminated to *m*/*z* 361 from probable H-C10-methyl migration to the extensive conjugated C11-carbonyl + H^+^. Further, unimolecular dissociations of *m*/*z* 377 from oleuropein-H^−^ could be intramolecular Ei-eliminations, pericyclic from its γ-H to carbonyls, then *m*/*z* 307 and *m*/*z* 275 as *syn*-eliminations of neutral 70 and 102 mws, respectively (Figure 3). Oleosidate-11-methyl ester at *m*/*z* 403, 2.1%, is also formed with neural semiquinone-like products. A gas-phase base-like hydrolysis generated a cascade of consecutive processes to *m*/*z* 223 aglycone and glucose followed by *m*/*z* 179 at very high energy. An identical pattern was recognized for oleuroside-H^−^ in ESI-MS/MS, dissociating to *m*/*z* 377, *m*/*z* 307, and *m*/*z* 275, with *m*/*z* 223 and *m*/*z* 179. This molecular dynamics of chimeric oleuropein model replicated the large site selectivity in base-catalyzed hydrolysis [36].

**Figure 4:**
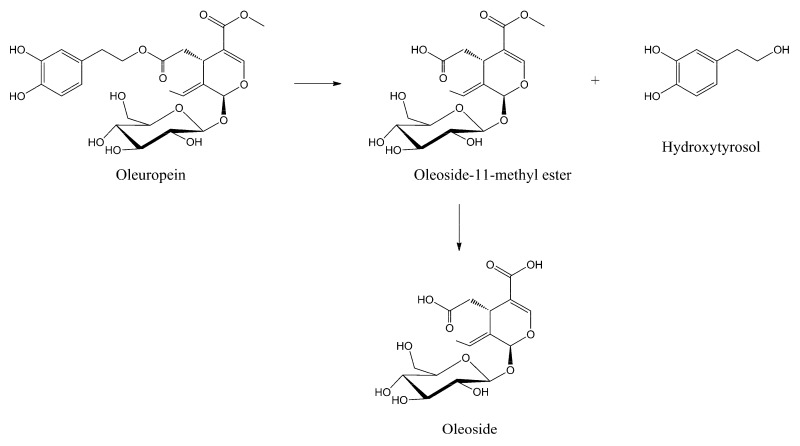
Polar hydrolytic sequence of oleuropein under basic catalysis, competing with radical processes.

The olive oil milling process, simulated under acid catalysis at pH = 4.2 for 120 min at 35 °C, provided intact oleuropein without any hydrolytic reaction of the two ester groups, preventing any oxidative degradation of the hydroxytyrosil to the corresponding *o*-quinone group followed by polymerization [31,37]. The BPseco remains almost intact under the table olive procedure, pH = 8.0 and 9.0 for 240 min at 25 °C. Thus, the BPseco change observed through table olive processing derives from microbiota action as lactic fermentation [38]. A complex degradation process of BPsecos is revealed at higher basicity at 25 °C (i.e., pH = 11.1, 12.8 and 13.2 for 120, 60 and 60 min, respectively). The formation of hydroxytyrosol and olesoside-11-methyl ester are found along with competing molecular dynamics at experimental pH = 12.7 for 40 min in nuclear magnetic resonance (NMR) mode [36]. However, complete hydrolysis on the methyl ester group at C-11 after further addition of base gave the oleoside as minor products (Figure 4) [39]. This process is governed by a large site selectivity, similar to the enzymatic degradation in olives drupes at the maturity stage, which avoids the apparent less hindered ester group of the BPseco chimeric structure [36].

The C11 ester carbonyl has not a simple electron distribution, but the highly extensive conjugation to the acetal O2 group is affected by inductive effect exerted by the glucoside oxygen [13]. Thus, the electron density effect on BPseco functionality, obscured the steric hindrance on C7 ester and determined the site selectivity under basic mode as well as highly acid resistance [36]. The true chimeric nature of the BPseco controlled the rate determining dynamics in activating the two major processes as polar and radical mechanisms. In polar mode, the base-catalyzed acyl-oxygen fission via a biomolecular reaction leads to the hydrolysis of oleuropein on the ester group at C7. In radical conditions, the C11=O reactivity competes with the pH controlled electron donor capacity of the catechol unit, thus resulting in aerial autoxidation.

**Figure 5:**
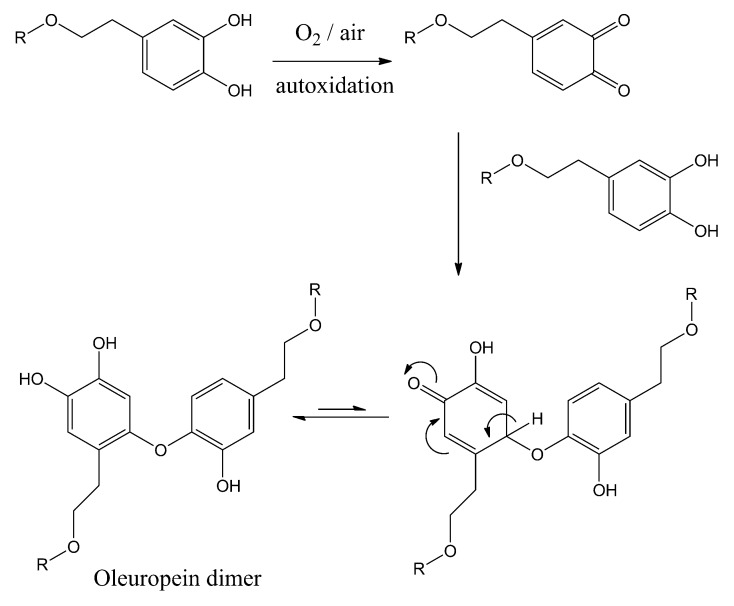
Reaction dynamics for the oligomerization of oleuropein under autoxidation, where *R* = Oleoside.

The radical autoxidation of the BPsecos and hydroxytyrosol was consistently observed above pH = 11.1, rising oleuropein dimers (Figure 5). A head to tail structure of the oligomerization mechanism takes place when polyphenol oxidase (PPO) is mixed with BPseco substrates under atmospheric O_2_ [40]. The initial transformation requires several steps leading to the *o*-quinone-like hydroxytyrosil residue from oleuropein after extended autoxidation, either by catalytic or enzymatic processes. This results in the oligomerization from addition reactions of oleuropein aromatic OHs on the enone of *o*-quinone-like moiety followed by tautomerisation to restore the aryl ring. The BPseco oligomerization could be the alternative pathway to the cyclization undergone by dimers of hyroxytyrosol in the peculiar methanoxocinobenzodioaxinone ring structure of the BP [41]. On the other hand, severe physico-molecular conditions were required for oleuropein hydrolysis associated with low yields, when 1 N H_2_SO_4_ at 100 °C or H_2_SO_4_ at pH = 0 for 3 h at 55 °C [42,43,44]. The reaction products were hydroxytyrosol, elenoic acid, hydroxytyrosil elenolate, and glucose. Significantly different environments were experienced for iridoid hydrolysis, lacking the dihydropyranyl arrangement [45,46]. However, the exact molecular dynamics of oleuropein is largely dependent on its structural heterogeneity towards polar and radical reactivity. Nevertheless, its biological properties should even be the direct consequence of the two major functionalities, secoiridoid and biophenol, the latter associated to its acidity dissociation constants and redox potentials.

On a thermodynamic basis, the autoxidation process of oleuropein and its BPseco analogs could be inefficient as it was ascertained at pH = 1.5 to 6.0. The exception operates at pH = 6.2, because of heavy metals and Cu contained in PPO enzyme. The enzyme activity increases with the highest amount of oleuropein during olive drupe ripening [47]. Nevertheless, BPsecos were very stable even at strong acidic pH [48]. Indeed, oleuropein and its BPseco analogs confirmed a high susceptibility to autoxidation at high pH values. The lower the one-electron reduction potential of BP semi-reduced free radical/reduced forms, the easier their oxidation under the O_2_/O_2_^−^ redox system. Therefore, the chimeric oleuropein preserves its original combomolecular structure towards important bioactive dynamics, under polar and free-radical modes [49]. In fact, oleuropein considered a quite evident simple acetal group, which appears very stable at specific acidic conditions or when mimic with natural β-glucosidase simulation [50]. In addition, H^+^ and H^·^ transfer shows a distinct behavior for BPs, latter associated with chain breaking dynamics with potent scavenger action on hydroxyl and superoxide radicals as well as peroxynitrite [51,52].

**Figure 6:**
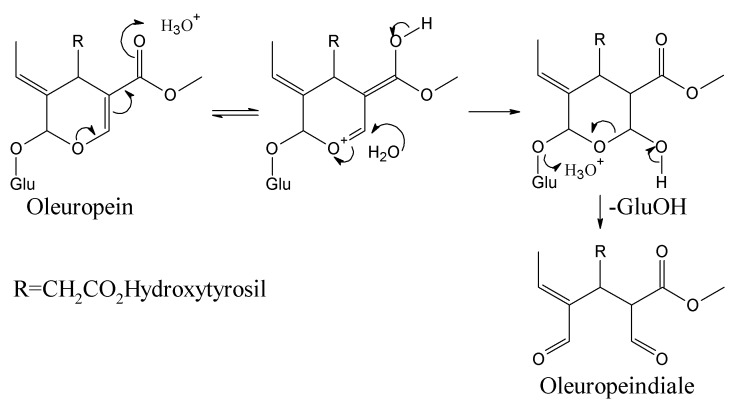
Acid-catalyzed hydrolysis of oleuropein.

BPsecos become phytoalexins when expressed by enzymatic reactions for the conversion to cyclic enones by PPO and anales as well as enales by β-glucosidase [13]. Esterases could intervene at olive drupes maturity stage with demethylation to demethyloleuropein at the carboxylate C11 and selective ester hydrolysis at the carboxylate C7 followed by further degradation to Cannizzaro-like metabolites and oleacein, reported as 3,4-DHPEA-EDA [2-(3,4-hydroxyphenyl) ethyl (3S,4E)-4-formyl-3-(2-oxoethyl)hex-4-enoate] [53,54]. After enzymatic processes, BPseco phytoalexin damages several macromolecules such as DNA and proteins, via alkylation and nucleophyle-carbonyl coupling. The nucleophilic dynamics at macromolecular reactive sites provides carbonyl oxygen loss to lysine alkylation, protein denaturation, and protein cross linkage [13]. The large resistance of oleuropein and its BPseco analogs towards specific acid-catalyzed hydrolysis requires a rational interpretation, which could be same structural effects that control their base-catalyzed reactivity. The oleuropein hydrolysis considers a simple reaction of its acetal-glucoside with the specific acid-catalyzed rupture of two carbon-oxygen bonds at C1 followed by water addition to the leaving glucose of BPsecos, which replaced by several proton-transfers originated from the basic site on C11=O (Figure 6) [55]. The overall dynamics involves a multistep pathway with pre-equilibrium of H_3_O^+^ and C=O base, external to the BPseco-ring and H_2_O linkage to C3-ring. A determining step follows by C1-OGlu bond cleavage at the transition state, leading to easy glucose released because of the resonance stabilized carboxonium at C1. Also, the concerted seco-ring opening to C1-enol, collapsing the dialdehyde form of oleuropeindiale and ligstrodidediale from oleuropein and ligstroside or to oleacein and oleocanthal from their demetylated derivatives, respectively.

The substitution pattern of the conjugated system O2-C3=C4-C11O_2_Me with its electron withdrawal of the seco-acetal group on O2 lowers the basicity of exo-OGlu. Thus, H^+^ transfer is incomplete at the transition state and explains the great decrease of reactivity experienced by BPsecos. Along the same direction, the increased stability on the corresponding C1 carbenium ion transient moiety is directly related to the hydrolysis rate of BPsecos. Therefore, the electron withdrawal from O2 occurring on the acetal OGlu of oleuropein decreases the equilibrium concentration of intermediate protonated on the same OGlu, making the departure of glucose as a leaving group less prone because of the further destabilization of eventual C1-carboxonium group. Therefore, the dynamics of proton transfer towards the basic site onto the C11=O carbonyl leads to the conjugate addition of H_2_O nucleophile on the α, β unsaturated carbonyl moiety of the BPseco ring as a 1,4-addition to β-C3 with the largest coefficient on the lowest unoccupied molecular orbital (LUMO) of oleuropein molecular system towards the thermodynamic product. This evolves with consequent bond cleavage for the novel approach to BPseco dynamics and to the bioactive hydrolytes found in green olives drupes and olive oil [56].

**Figure 7:**
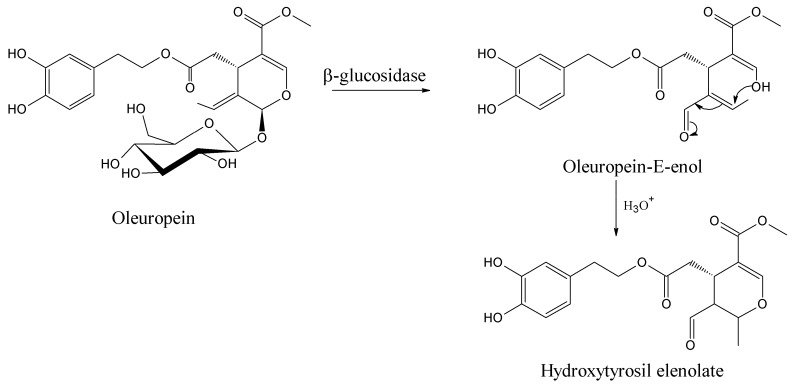
The cascade dynamics of oleuropein to hydroxytyrosil elenolate in concert under acid-catalyzed conditions.

This dynamic mechanism is far from a simple step-by-step transformation of oleuropein starting material to its hemiacetal on C3 and to oleuropeindiale under acid-catalyzed reactions [57,58]. The development of a cascade conversion in concert provides the recovery of hydroxytyrosil elenolate without any possible isolation of intermediates [43]. These BPsecos are reactive open cycle species rapidly undergoing ring closure via a Michael-type process to the six-membered structure hydroxytyrosil elenolate (Figure 7). It is one of the most abundant BPseco derivatives found in olive waste water after acid hydrolysis [59]. However, the rate of cyclization depends on their substituents. For instance, the lack C11-carboxymethyl in oleacein from demethyloleuropein or the π-bond on C9 shifted to C8 in oleuroside slows down the overall dynamics to elenolate ring formation, since the electron withdrawn exerted by the C11-carboxymethyl lessens the nucleophilic attitude of C3-enol [2,6,60,61]. Therefore, the acetal hydrolysis of oleuropein at its seco-site is easy and rapid exclusively when activated by its native β-glucosidase [62].

## 3. Computational Mapping of Oleuropein

**Figure 8:**
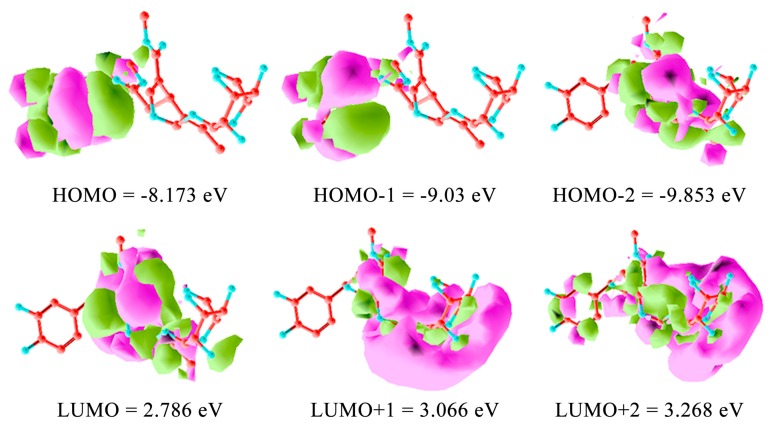
Oleuropein orbital surfaces and energy levels for highest occupied molecular orbital (HOMO), HOMO-1, HOMO-2, and lowest unoccupied molecular orbital (LUMO), LUMO+1, LUMO+2 using ab initio calculation with the 6-311G(d,p) basis set. Isosurface of the HOMO and LUMO consists of two colors (green and pink), which represents the positive and negative isosurfaces. The choice of color for positive or negative part is arbitrary.

For computational mapping of oleuropein, the ab initio semi-empirical self-consistent-field (SCF) hartree-fock HF calculation was carried out employing 6-311G(d,p) basis set. Geometry was optimized at the HF/6-311G(d,p) level. The coefficients of the frontier molecular orbitals were obtained from the HF/6-311G(d,p) optimized geometries. The 6-311G(d,p) generated highest occupied molecular orbital (HOMO) and LUMO molecular orbitals of 1–3. The ab initio method with the HF/6-311G(d,p) geometrical optimization is more accurate than Austin model 1 (AM1), which uses a minimal basis set of valence Slater type s and p in an atomic orbital to extend valence-electron molecular orbitals [17]. Ab initio methods are better for energetics evaluation. The remarkable molecular framework distributes HOMO and HOMO-1 for radical processes and HOMO-2 for electrophilic site. LUMO is clearly the polar molecular dynamics, devoted to the nucleophilic reaction (Figure 8). At pH = 7.0, oleuropein unveils a very low dissociation (0.2%) according to calculated values for its p*K*_a1_ = 9.70 ± 0.10, which involves the 3-OH on the aryl group as performed adopting the Hammett-type equation. This allowed utilization of apparent p*K*_a_ values, thus mimicking the experimental order of deprotonation in the hydroxytyrosol residue of oleuropein in water solution [63,64]. The earlier p*K*_a1_ = 9.07 ± 0.02 and p*K*_a2_ = 9.98 ± 0.06 showed for catechol were 9.25 and 13.0, respectively [30,65]. Calculated p*K*_a2_ gave then 12.52 ± 0.20 for 4-OH aryl, which renders oleuropein barely mono-deprotonated in the neutral environment with p*K*_a3_ = 12.80 ± 0.70 at 2’, p*K*_a4_ = 13.54 ± 0.70 at 4’, p*K*_a5_ = 14.48 ± 0.10 at 6’, and p*K*_a6_ = 14.81 ± 0.70 at 3’on the glucose moiety of oleuropein. The mono-deprotonated oleuropein-H^−^ generated during the ESI-MS localized the negative charge on the 3-aryloxy site as an alternative to the p*K*_a4_ = 13.54 ± 0.70 at 4’ which is similar to verbascoside [30,66,67]. The two OH aryl groups at 3 and 4 positions of the 3,4-dihydroxyphenyl residue are not equivalent as in the catechol molecule. This is shown by the SCF molecular orbital calculations on hydroxytyrosol, which was optimized at the AM1 level where the highest positive excess charge accumulated on the H bonded to O at 4 in the hydroxytyrosol ring [51]. This specifies, the primary transfer attitude due to a free radical dynamics of its main bioactivity, i.e., the scavenging of reactive oxygen and nitrogen species, the major cause of several human degenerative diseases.

The BPseco offered its bident functionality of ester site to the alternative release of hydroxytyrosol and methanol from position C7 and C11 respectively. Thus, the longstanding conjugation is from O2 until C11=O changed the overall dynamics of carbonyl esters and acetal groups because C11-ester differs from the C7 as electronic and steric structures. The same happened to the acetal group, which was not a simple but a conjugated system. The combo-functionality of BPseco provided perturbated molecular orbitals due to reciprocal conjugation represented at HOMO −8.173 eV and LUMO 2.786 eV calculated with ab initio SCF and 6-311G(d,p) basis set (Figure 8). Ab initio computational methods are quite reliable for small and medium-sized molecules. Semi-empirical molecular orbital shown −8.669 eV, and, −0.111 for HOMO and LUMO, respectively. The HOMO value is essentially the same, while the LUMO is highly underestimates by the semi-empirical method AM1. Semiempirical AM1 underestimate the normal component of the polarizability tensor, the first hyper polarizability tensor strongly depends on the electronic structure of BPsecos. As a consequence, the HOMO-LUMO calculations are affected. The observed HOMO-LUMO gap is 5.387 eV, which means the BPseco absorbs in the UV. Oleuropein molecular dynamics is rationalized by HOMO electron distributions, thus the low reactivity of oleuropein under strong acid catalysis is prevented, requiring the general acid-base environment. Specifically, the acid-base catalysis for the H^+^ induction of hydrolytic reaction on BPsecos is in competition between C11=O and the forbidden site on the hydroxytyrosil moiety unreactive under this condition being only available for electrophilic aromatic substitutions. Therefore, the reaction rate is not dependent only on pH but is also a function of H^+^ buffer concentrations. The buffer aids in stabilizing the transition state via donation of H^+^ onto C11=O. The reaction rate depends on the buffer concentration as well as on the appropriate H^+^ site at the transition state. The largest coefficient of conjugated system in oleuropein LUMO resides on the C3, the soft β-carbon of the α-β unsaturation of BPseco. Thus, the HO^-^ nucleophile attack occurs at C7=O site because the electrostatic control is dominant due to the lowest charge density of its LUMO. Therefore, both HOMO and HOMO-1 on the hydroxytyrosil moiety, which appear to be highest in their energy states compared to HOMO-2. Oleuropein reveals its first facile dynamic behavior toward radical reaction as free radical quencher and then as polar electrophiles through carbonyl groups on the newly formed aldehydes and *o*-quinone-like moiety. This key orbital interaction establishes the start-up step for BPseco hydrolysis leading to the fission of combo-oleuropein, which lose its functional complexity responsible for the radical reactivity and leaving alone the molecular structure for the polar reaction.

## 4. Conclusions

The quality olive oil and table olive production involves complex BPsecos molecular dynamics. Therefore, understanding the molecular mechanism of oleuropein and its analogs such as ligstroside, demethyloleuropein and oleuroside along with their bioactives oleacein, oleocanthal and elenolates could improve the olive processing and functional food characteristics of bitterness, pungency, and astringency. Overall, the native enzymatic reaction cascade lasted a short time, which is different from acid-catalyzed mechanism. The olive oil extraction from drupes using crushing and malaxing process for 15–90 min at 25–35 °C, reveals a sufficiently large reduction of catechol-like BPs to 70% due to their oxidation by PPO. Indeed, temperature negatively influences quality attributes. HOMO and LUMO computational mapping provide molecular dynamics for bioactives coming from oleuropein and pave the way to further experiments on nutrigenomics. Thus, good manufacturing practice is part of the quality control system, which offer enormous health beneficial rich biomolecules containing olive oil and table olives for advanced functional foods and nutraceuticals.

## Abbreviations

AM1	Austin model 1
BPs	Biophenols
BPsecos	Biophenols and secoiridoids
CAD	Collision activated dissociations
FAB	Fast atom bombardment
HF	Hartree-fock
HOMO	Highest occupied molecular orbital
HPLC-ESI-MS	High performance liquid chromatography electron spray ionization mass spectrometry
IRESMO	Istituto di ricerche europee in scienze molecolari
H_2_SO_4_	Sulphuric acid
LUMO	Lowest unoccupied molecular orbital
MS	Mass spectrometry
MEMEGNMR	Molecular ecology, microbial ecology and evolutionary geneticsNuclear magnetic resonance
OGlu	Glucose
PPO	Polyphenol oxidase
SCF	Semi-empirical self-consistent-field
